# Continuum Molecular Simulation of Large Conformational Changes during Ion–Channel Gating

**DOI:** 10.1371/journal.pone.0020186

**Published:** 2011-05-20

**Authors:** Ali Nekouzadeh, Yoram Rudy

**Affiliations:** Cardiac Bioelectricity and Arrhythmia Center and Department of Biomedical Engineering, Washington University in St. Louis, St. Louis, Missouri, United States of America; Memorial Sloan Kettering Cancer Center, United States of America

## Abstract

A modeling framework was developed to simulate large and gradual conformational changes within a macromolecule (protein) when its low amplitude high frequency vibrations are not concerned. Governing equations were derived as alternative to Langevin and Smoluchowski equations and used to simulate gating conformational changes of the Kv7.1 ion-channel over the time scale of its gating process (tens of milliseconds). The alternative equations predict the statistical properties of the motion trajectories with good accuracy and do not require the force field to be constant over the diffusion length, as assumed in Langevin equation. The open probability of the ion–channel was determined considering cooperativity of four subunits and solving their concerted transition to the open state analytically. The simulated open probabilities for a series of voltage clamp tests produced current traces that were similar to experimentally recorded currents.

## Introduction

Molecular dynamics (MD) simulations is a method used to model the molecular motion of proteins [1], including ion channels [2]. Simulations are conducted by solving the equations of motion for all atoms of a protein, starting from their known initial locations and assigned random initial velocities. The resultant motion is a high frequency, low amplitude vibration of the protein atoms. MD had limited success in predicting the large and gradual conformational changes that underlie the physiological function of many proteins (e.g. ion–channel gating). MD simulations are massive and usually can simulate up to 1 microsecond of protein dynamics and in some cases up to 1 millisecond using special computer architectures [3], while the large conformational changes of ion–channels (and other proteins) occur over tens of milliseconds. Due to the very large number of degrees of freedom, it is impossible to analyze the motion in the entire configuration space. Therefore, MD generates a trajectory of conformational changes associated with assigned initial conditions for the atoms. To facilitate computations, MD simulations may be influenced to drag the structure toward preferred conformations by reducing the potential energy of those conformations [4], [5]. Virtual increase of temperature to facilitate the passage of the structure through local minima has also been considered [6], as well as grouping atoms into coarse grains to reduce degrees of freedom [7]. With these manipulations, the motion trajectory samples a broader region in configuration space. The estimates for the potential of mean force [8], computed this way, rely on how accurate the motion trajectory represents the entire space of trajectories. Clearly, simulating with MD the microsecond dynamics of protein in a statistically meaningful manner is challenging, whereas simplified models allow one to study the millisecond dynamics in a computationally efficient way.

Large conformational changes usually involve gradual dislocations of protein segments which can be modeled with a limited set of degrees of freedom, *x_j_*. These degrees of freedom are usually translation and rotation of protein segments with reinforced secondary structure (e.g. helices). The proposed modeling framework of this paper simulates the protein dynamics within the entire configuration space of large conformational changes (all combinations of *x_j_*), without explicitly simulating high frequency vibrations of single atoms and thermodynamic properties. It simulates the average conformational changes over *Δt*, a time window sufficiently larger than the time between collisions. In this study we analyzed the molecular motion of particles under the influence of a conservative force field using a kinematic theory approach and derived the governing equations of the motion. These equations are more accurate alternatives to Langevin equation and Smoluchowski equation which have been used to model the gradual motion of proteins in a reduced configuration space, neglecting its atomic vibrations [9], [10], [11].

According to Newton's second law of motion, velocity of a particle (referred to as a target particle in this paper) on a molecular scale is determined by:
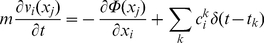
(1)where *m* is the mass of the target particle, *v_i_* is its velocity along coordinate *x_i_*, Φ is the potential of any existing conservative force field, and the last term on the right represents the stochastic force of thermodynamic collisions on the particle. *t_k_* is a time when another particle (referred to as a colliding particle in this paper) hits the target particle and 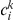 is the impact momentum transferred to the target particle during the collision. The thermodynamic forces on the target particle have a non–zero mean that is proportional to the target particle velocity (in opposite direction). Therefore, Newton's equation assumes the form of Langevin equation:
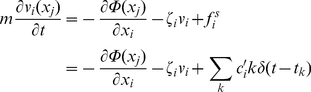
(2)where 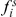 is a zero mean stochastic force and 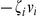 is a friction force that models the non–zero mean of the thermodynamic force.

However, modeling the non–zero mean of the stochastic force by the term 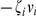 alters the governing equation and consequently the stochastic properties of the resultant velocity. Assume the simplest case in the absence of a conservative force field. The actual motion of the target particle consists of constant velocity motions between any two consecutive impacts. At any collision incident, the velocity of the target particle changes abruptly by 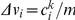 to a new random velocity. The expectation value of the velocity after the collision is a constant fraction of the velocity before the collision. But according to the Langevin equation, after any collision the expectation value of the velocity equals its velocity before the collision, and the magnitude of velocity decreases exponentially between collisions (rather than being constant). It means that Langevin equation overestimates the velocity magnitude right after a collision, and then compensates for this overestimation by reducing it toward zero between collisions. Therefore, the velocity ensembles that result from solving the Langevin equation are different than the actual velocity ensembles and consequently the Langevin equation may not be an accurate choice for determining the statistical properties of the motion (e.g. probability density function and autocorrelation of the velocity), or for estimating the motion trajectories when the stochastic motion is significant. An illustrative example of these Langevin equation properties is provided in the Text S1.

In Langevin model of motion, the stochastic force is assumed to be a zero mean Gaussian process with a Dirac delta autocorrelation function. Based on these assumptions, the variance of the stochastic force can be determined in terms of the variance of the velocity distribution using the fluctuation-dissipation theorem [12]. However, the prediction of Langevin equation for the velocity, in response to a stochastic force with a Gaussian distribution, does not have a Gaussian distribution (Figure S2 in Text S1); this contradicts the well known Boltzmann–Maxwell distribution (a Gaussian distribution) for the velocity (Figure S4 in Text S1). Compared to a Gaussian distribution with the same variance, the velocity distribution in Langevin equation has higher densities for velocity magnitudes in close vicinity of zero and for large velocity magnitudes (Figure S2 in Text S1). Because the friction term causes a decay of the velocity magnitude toward zero between the collisions (Figure S1 in Text S1), the probability of velocity magnitudes close to zero is increased (compared to the Gaussian distribution). And because the Langevin equation overestimates the velocity magnitude after a collision (Figure S1 compared with Figure S3) the probability of having large velocity magnitudes is increased as well. In addition, application of the Langevin equation is constrained to external force fields that are almost constant over the diffusion length.

Motivated by the need for a model that can accurately replicate the velocity distribution and does not constraint the conservative force field as in the Langevin equation, we introduce here a new modeling framework that is applicable to large, gradual conformational changes of a protein. We analyze the actual motion of a particle (protein segment) that undergoes multiple collisions in a probabilistic domain (a kinetic theory approach) and derive a governing equation for the average velocity. Unlike the Langevin equation, the governing equation of the average velocity does not have an inertial term. It should be emphasized that the inertial term is not neglected assuming a large friction coefficient (as assumed in the high friction limit of Langevin equation), it simply does not appear in the governing equation of the average motion after applying Newton's law of motion for a system with multiple collisions. Because during the conformational changes of a protein segment the conservative force may vary significantly over the diffusion length, we do not consider it constant in our analysis and derive a more general equation for the effect of a conservative force on the motion trajectory and the probability distribution. The stochastic term in our equation appears as stochastic velocity (rather than force) and does not have a delta autocorrelation function. Note that the autocorrelation of the stochastic force is assumed to be a Dirac delta function in deriving the statistical properties of motion from Langevin equation and in deriving the Einstein–Smoluchowski relation between the friction coefficient and the diffusion constant. In reality, the autocorrelation decreases gradually over *Δt*.

The newly developed modeling framework is used to simulate the conformational changes of the voltage sensor region (S1 to S4) of the Kv7.1 ion–channel during gating and the resultant open probability is compared to experimentally recorded macroscopic currents.

## Results

We develop a model for the gradual motion of the helical transmembrane segments of an ion–channel protein. We use this model to simulate the motion trajectories and the transient probability distribution in the configuration space. Further, we use the results of these simulations to compute the single channel and macroscopic currents carried by the ion–channel. The model developed in this paper represents the stochastic motion of a particle (here a protein segment) on a molecular scale. It consists of two key sets of equations: equations (38)–(40) and equations (43), (47)–(49). The first set governs the average motion (over a time window) of the particle and can be used to simulate the motion trajectories. Compared to Langevin equation, a key feature of this equation set is that it accounts for potential fields that are not constant over the diffusion length by including the higher derivatives of the potential function. Additionally, it provides more accurate estimates for the stochastic properties of the motion. The second set of equations (derived based on the first set) governs the transient and steady state distribution of the particle in its configuration space. These equations also account for potentials that are not constant over the diffusion length.

For simulating the structural dynamics and electrophysiological function of the ion–channel, the first equation set is used to generate motion trajectories and from those the single channel current traces. The second equation set is used to compute the channel open probability and the macroscopic current through a large ensemble of ion channels.

### Equation of Motion for a Protein Segment

**Figure 1:**
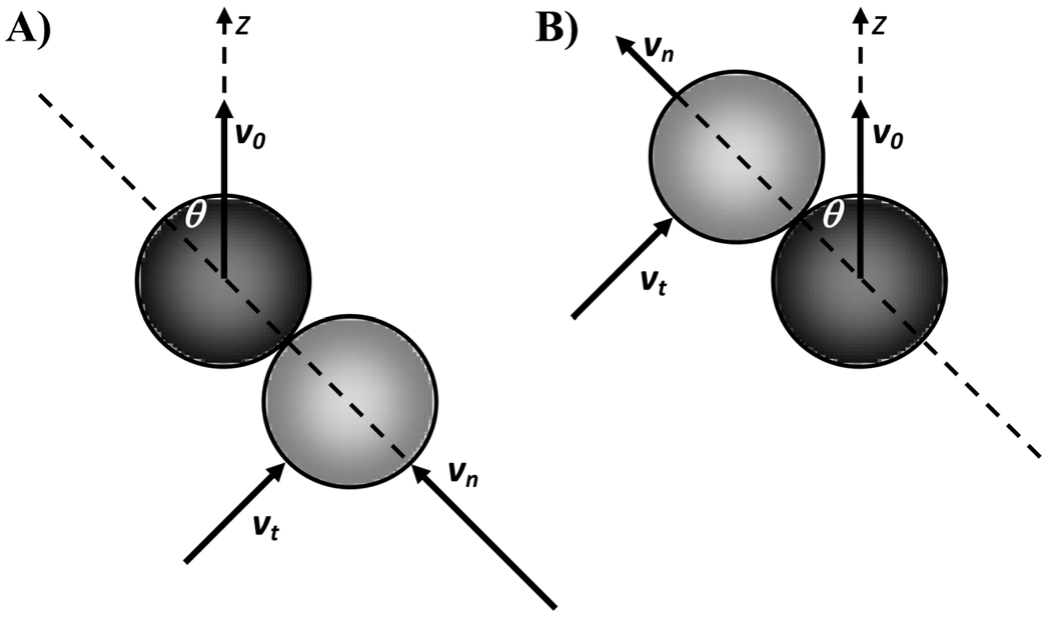
Collision between a colliding particle (light gray) and the target particle (dark gray) along an arbitrary direction of impact (dashed line). The colliding particle can have any velocity for an impact along any direction. Panel A shows a collision when the velocity of the colliding particle along the impact direction (***v_n_***) is faster than the velocity of the target particle along this direction. In panel B, the velocity of the colliding particle is slower.

The stochastic motion of a target particle (representing a protein segment) and colliding particles are considered in 3D Cartesian space. Because of symmetry, the velocity of the colliding particles is uniformly distributed in all directions. Also, the probability that a point in space is occupied by a colliding particle is equal everywhere. Consider a target particle moving with a velocity *v_0_* in the space shown in **Figure 1** where the *z* direction is chosen along the velocity. Assume an arbitrary location on the surface of the target particle that may be hit by a colliding particle. Panel A shows the impact point and the relative position of the target particle and the colliding particle. An impact occurs if the velocity of the colliding particle along the impact direction (dashed line) is larger than the velocity of the target particle along this direction: 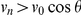. The tangential velocity of the colliding particle, *v_t_*, can have any value. For a collision at an opposite location (panel B), the condition of impact is: 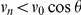. Therefore, along any impact direction the colliding particle may have any velocity. This means that the probability distribution of the impact direction, the colliding particle velocity and consequently the time and distance between the collisions are independent of the target particle velocity. The impact direction may be quantified by angles *θ* (between 0 and π/2) and *ϕ* (between 0 and 2π) in a spherical coordinate system. Particles with equal mass exchange their component of velocity along the impact direction. Therefore, 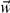, the velocity of the target particle after the impact is:
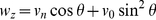
(3)

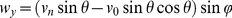
(4)

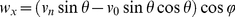
(5)where *θ* and *ϕ* determine the direction of the impact line. Because the probability of occupying any location in space is uniform, the probability distribution of the impact line is uniform. The probability distribution of 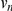 is the same as the probability distribution of the velocity component along any axis (i.e. *x*, *y* or *z*), which is known to be a Gaussian distribution. The expectation value of the target particle velocity after the impact is:
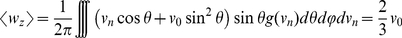
(6)

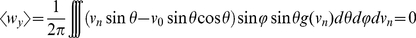
(7)

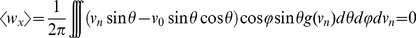
(8)where 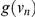 is the probability density function of a 1D component of the velocity. It should be emphasized that 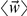 is independent of 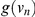. The expectation of the velocity in the *z* direction is non–zero, meaning that the stochastic velocity of a particle is not memory–less and therefore its autocorrelation function is not a delta function. However, this value vanishes after about ten impacts, implying that the autocorrelation approaches zero after a time required for about 10 impacts.

If there is no conservative force field acting on the particle, it will have a zero mean normally distributed stochastic velocity, 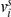. The conservative force will add a deterministic velocity, 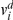 to this zero mean stochastic velocity (causing the resultant stochastic velocity of the particle to have a non zero mean):
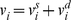
(9)Assume that 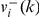 is the velocity of the particle right before the *k^th^* impact, 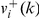 its velocity right after the *k^th^* impact and 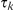 is the time interval between the *k^th^* and (*k*+1)*^th^* impacts. Between the *k^th^* and (*k*+1)*^th^* impact, the particle accelerates under the influence of the conservative force field and as a result, its deterministic velocity increases by 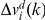 right before the (*k*+1)*^th^* impact is:
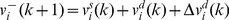
(10)During an impact, two particles exchange some (or all, if they have equal masses) of their momentum in the direction of impact. Depending on the direction of impact and mass of the particles, the particle loses a fraction of its velocity and gains a fraction of the velocity of the colliding particle:
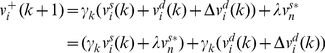
(11)where 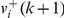 is the velocity of the particle immediately after the (*k*+1)*^th^* impact, 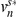 is the normal component of the velocity of the colliding particle and *γ_k_* is the fraction of particle velocity that is preserved during the impact. If there is no conservative force field, there would be no deterministic velocity and the velocity after the (*k*+1)*^th^* impact is:
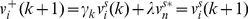
(12)From a statistical perspective, the stochastic velocity depends on the probability of collision, and on the probability distribution of the impact direction and the colliding particle velocity. These statistical parameters are independent of the velocity of the target particle and consequently its deterministic part. Therefore, the stochastic component of the velocity would be the same with or without conservative force field, and we may conclude that:
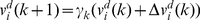
(13)Note that 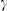 is a random variable that obtains random values according to its probability distribution at each collision. The magnitude of 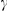 is always less than 1. The deterministic velocity can be calculated as:
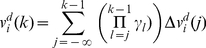
(14)where *j* refers to all collisions prior to the *k^th^* collision. The right side of equation (14) is a convergent series. Note that although we used the notion of infinity, in practice the series approaches a constant value if we calculate it up to a few preceding collisions (∼10 for equal mass particles).

To find the global velocity of the particle we average the velocity over a time interval *Δt*. The time interval is chosen sufficiently long, such that the average of a stochastic parameter over *Δt* closely approximates its expectation value, and sufficiently short such that gradual conformational changes are small during *Δt*. 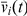 is defined as the average of the particle velocity over this time window:
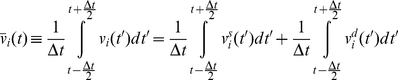
(15)We define 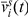 as the stochastic component of the average velocity. It is the average of the stochastic component of velocity, 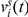:
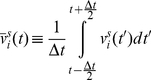
(16)

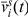
 is a zero mean stochastic process with a smaller variance compared to 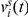. Note that the autocorrelation of 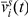 vanishes for times beyond *Δt*. Autocorrelation of 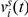 may be considered a delta function compared to 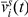 because *Δt* is orders of magnitude longer than the time of several collisions.

The average of the deterministic component of velocity is:
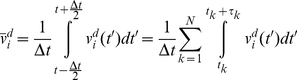
(17)where *N* is the total number of collisions during *Δt*. Between the *k^th^* and (*k*+1)*^th^* impact, the particle travels between *x_i_*(*k*) and *x_i_*(*k*+1) and is subjected to a conservative force *f_i_*(*x_j_*) (per unit mass). The global motion of the protein segment (target particle) during *Δt* is associated with the deterministic velocity. *Δt* is assumed sufficiently small such that the conservative force field can be considered constant over the associated global displacement. However, during *Δt* the range of motion (diffusion length) depends on the stochastic velocity that is much larger than the deterministic velocity and consequently the particle travels much farther (than the global displacement) in both the positive and negative directions. The conservative force may or may not be constant over this range. Note that *Δt* needs to be sufficiently large, such that the time average and the ensemble average are the same (e.g. *Δt* includes at least 100 collisions). Therefore, we assume that *f_i_*(*x_j_*) is not constant over the diffusion length, 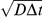, and can be approximated accurately by several terms of its Taylor expansion.
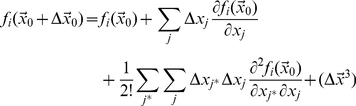
(18)Where 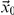 is the location of the particle at the middle of time window. The number of required terms depends on the variation of the force field. Between the *k^th^* and (*k*+1)*^th^* impacts we may write:
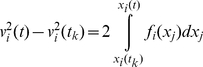
(19)where *t* is between *t_k_* and *t_k_*_+1_. Substituting for 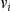 from equation (9) and assuming that 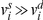 we may rewrite equation (19) as:
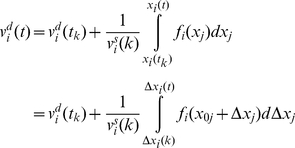
(20)where 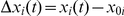.

**Figure 2:**
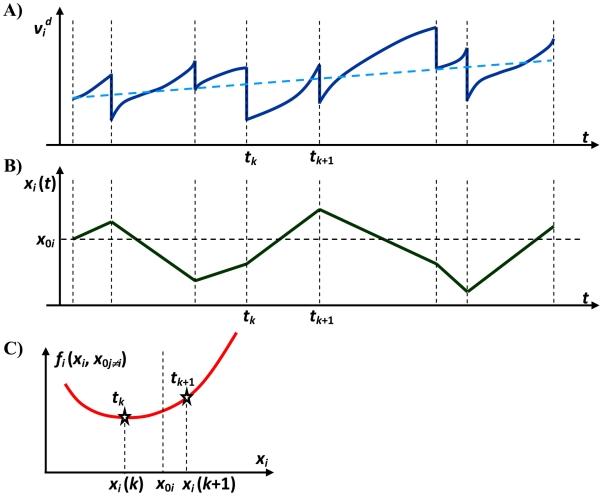
A schematic presentation of the deterministic velocity and location of a particle along *i^ th^* direction. Expectation value of deterministic velocity (dashed blue line in panel A) varies gradually over time. Location varies linearly between any two consecutive collisions (panel B). The traveled distance between any two consecutive collisions is sufficiently long that the conservative force (panel C) may not be considered constant along the path.

Note that the motion is a multidimensional motion. Equation (20) integrates over the *x_i_* direction, but the location of the particle (*x_j_*) varies in all directions. Because 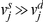 and 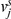 is constant between impacts we may write:
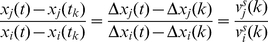
(21)and consequently:
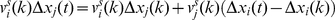
(22)where *i* and *j* refer to any two arbitrary degrees of freedom and 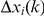 refers to 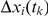. Substituting *f_i_* with its Taylor expansion in equation (20) and substituting for 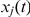 from equation (22) we may write:
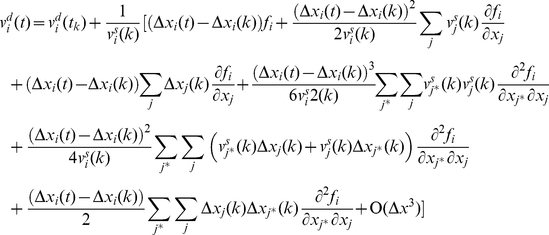
(23)Using equation (23), the 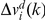 that is defined in equation (10) is:
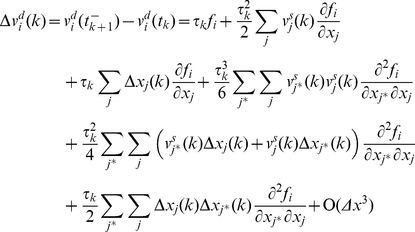
(24)And the average of the deterministic component of the velocity is:
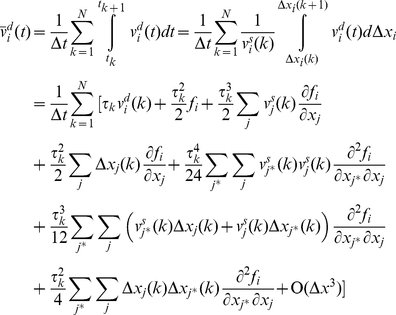
(25)

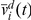
 depends on the random variables 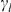, 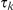 and 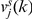 and therefore has fluctuations. However, these fluctuations are very small compared to 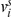 and may be incorporated in the stochastic velocity component. From a thermodynamic perspective this negligible addition to the stochastic velocity represents the very small addition to the particle temperature during Δ*t* as a result of friction. **Figure 2** provides a schematic presentation of the 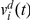 and 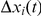. Although the deterministic velocity has some fluctuations, its expectation value varies gradually over time (panel A) and causes a gradual global motion of the particle. The large amplitude stochastic velocity (compared to deterministic velocity) causes the particle's location to vary linearly between any two consecutive collisions (panel B). We assume that the traveled distance between any two consecutive collisions is sufficiently long such that the conservative force cannot be considered constant along the path (panel C).

We define the global deterministic velocity as the expectation value of equation (25) :
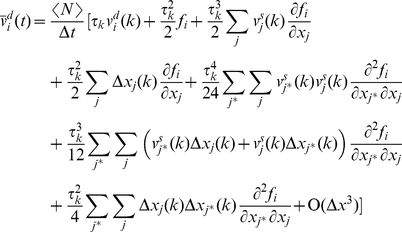
(26)Where *k* refers to a collision incident in the middle of a time window 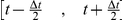 and the expectation values of *f_i_* and its derivatives should be calculated at *x_j_*(*t*), the expectation location of the particle at time *t*. *γ* and *τ* are uncorrelated stochastic processes and are independent from each other and from 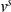. 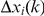 is independent of 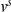, *γ* and *τ*. Considering that the motion is symmetric in the positive and negative directions we may write:
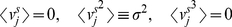
(27)

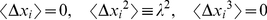
(28)

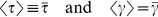
(29)And the equation (26) can be simplified to:
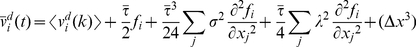
(30)The expectation value of 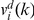 can be determined using equations (14) and (24) as:
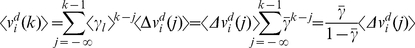
(31)

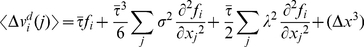
(32)It should be mentioned that because of the symmetry of the motion (location and velocity) in both the negative and positive directions, all the odd derivatives of *f_i_* will be eliminated. Combining equations (30), (31) and (32), the deterministic component of the velocity is:
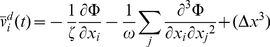
(33)Where:
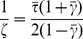
(34)

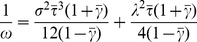
(35)

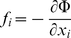
(36)And the equation of motion for the average velocity is:
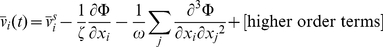
(37)In general, the characteristic parameters of the motion (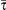, 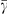, 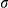 and 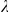) are different for different degrees of freedom and we write equation (37) in a more general form:
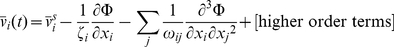
(38)where
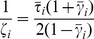
(39)

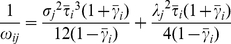
(40)


### Probability Distribution of the Segment in Configuration Space

Time variation of the probability distribution is equal to the negative divergence of the probability flux. If the velocity field is a time invariant process, then the probability flux is the expectation value of the multiplication of the velocity field and the probability distribution. Therefore:
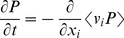
(41)For a stationary velocity field:
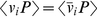
(42)Using equations (38) and (42) we may rewrite equation (41) as:
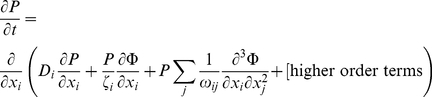
(43)The stochastic velocity leads to a diffusion (in the probability distribution) with a diffusion constant *D_i_*. At steady state, the time derivative of the probability distribution is zero:
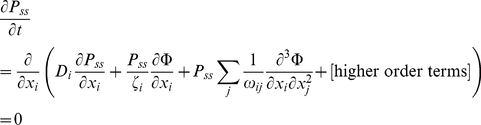
(44)This means that the total flux leaving a differential element in configuration space is zero. Imposing the principle of detailed balance (microscopic reversibility), probability flow in all directions of the configuration space should be zero (no circulation in probability space):
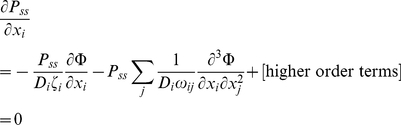
(45)In order for *P_ss_* to exist, its partial differentials in equation (45) must present a total differential, meaning that for any *i* and *k*:
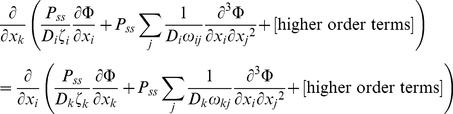
(46)Equation (41) is satisfied if and only if:
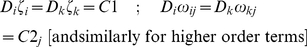
(47)where *C*1 and *C*2*_j_* are constants that vary with temperature. Therefore, the principle of detailed balance requires that the terms 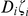 and 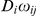 retain the same value for all *i*. Under this condition the steady state distribution of probability has the following closed form:
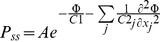
(48)

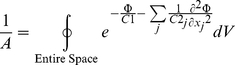
(49)


### Simulation Results for Conformational Changes of the Kv7.1 Ion–Channel Voltage Sensor during Gating

#### Degrees of Freedom

**Figure 3:**
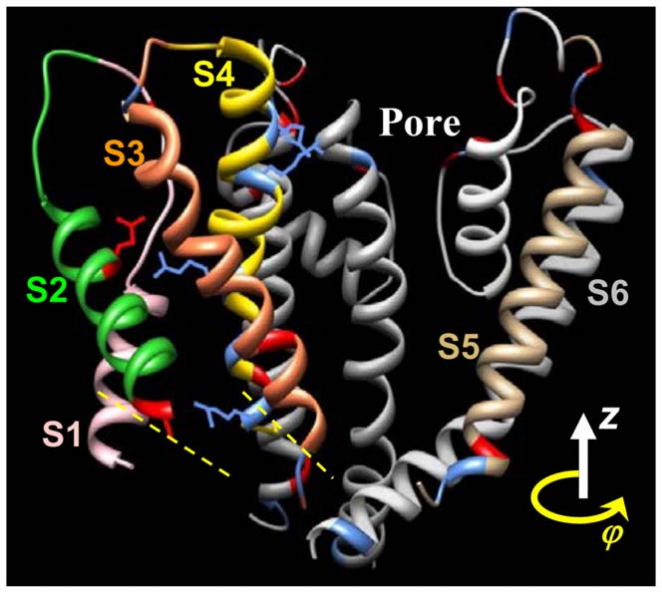
3D structure of Kv7.1 (one subunit) and its transmembrane helices in the open conformation. The structure was computed based on homology with Kv1.2 using its known crystal structure [13]. Motion of the S4–S3 complex is assumed to be the major conformational change during channel opening and closing (gating). The loop connecting S2 to S3 and the linker connecting S4 to S5 are not shown (dashed lines). Dark gray helices are S5 and S6 of the neighboring subunit. Red segments are negatively charged residues and blue segments are positively charged residues.

A 3D structure of the Kv7.1 channel was derived previously [10], [11] based on homology to Kv1.2 with a known crystal structure in the open state [13] (**Figure 3**). To simulate the protein structural dynamics, we first identify the significant degrees of freedom for large conformational changes during gating. A voltage dependent potassium channel consists of four similar polypeptides (subunits), each includes six transmembrane segments termed S1 to S6. S5 and S6 form the pore and S1–S4 form the voltage sensor that undergoes large conformational changes in response to variations in membrane potential. Voltage sensors of the four subunits are sufficiently distant so that their conformational changes can be assumed independent, except for the last transition to the open state that requires a cooperative transition of all four voltage sensors [14], [15]. Within each voltage sensor, transition and rotation of S4 and probably S3 are associated with channel gating [16], [17]. The relative location of S3 and S4 is constrained by the very short loop that connects them on one side and a salt bridge between S4 Histidine H240 and S3 Aspartic Acid D202 on the other side. Therefore, we assume that the significant degrees of freedom that can closely model the conformational changes during gating are translation and rotation of S4 and S3 as a single complex. Translation is perpendicular to the membrane surface, positive outward from the cell (*z* direction); rotation is about an axis parallel to the first principal direction of the S4-S3 complex, located 5 Angstrom away from its center. The axes of rotation and translation were chosen to comply with the geometrical constraints imposed by the S4-S5 linker and to reduce the probability of a steric overlap of the S4-S3 complex with neighboring segments. Based on our recent loop closure technique [18], we found that the loop between the S3 and S2 segments is sufficiently large not to impose significant geometrical constraint. Based on experimental data [19], [20], we allow S4-S3 to move outward up to 6 angstrom and inward up to 14 angstrom (in 0.2 angstrom steps) and rotate 1 radian in the clockwise and counter clockwise directions (in 0.05 radian steps).

#### Energy Landscape

Energy landscapes were constructed at multiple membrane potentials by computing the total electrostatic potential of a voltage sensor at all the conformations it assumes in the configuration space described above. The transmembrane potential adds a uniform electric field to the dielectric region (protein and membrane); it approaches zero outside this region. The potential energy of steric interactions was modeled with the Lennard–Jones potential [21]. Axis of rotation was adjusted by trial and error to eliminate any steric clashes between backbone atoms and also major clashes between side chain atoms. A penalty function was also applied for outward and inward motion of S4–S3 beyond 4 and 8 Angstroms respectively, to mimic the geometrical constraint imposed by the S4–S5 linker.

**Figure 4:**
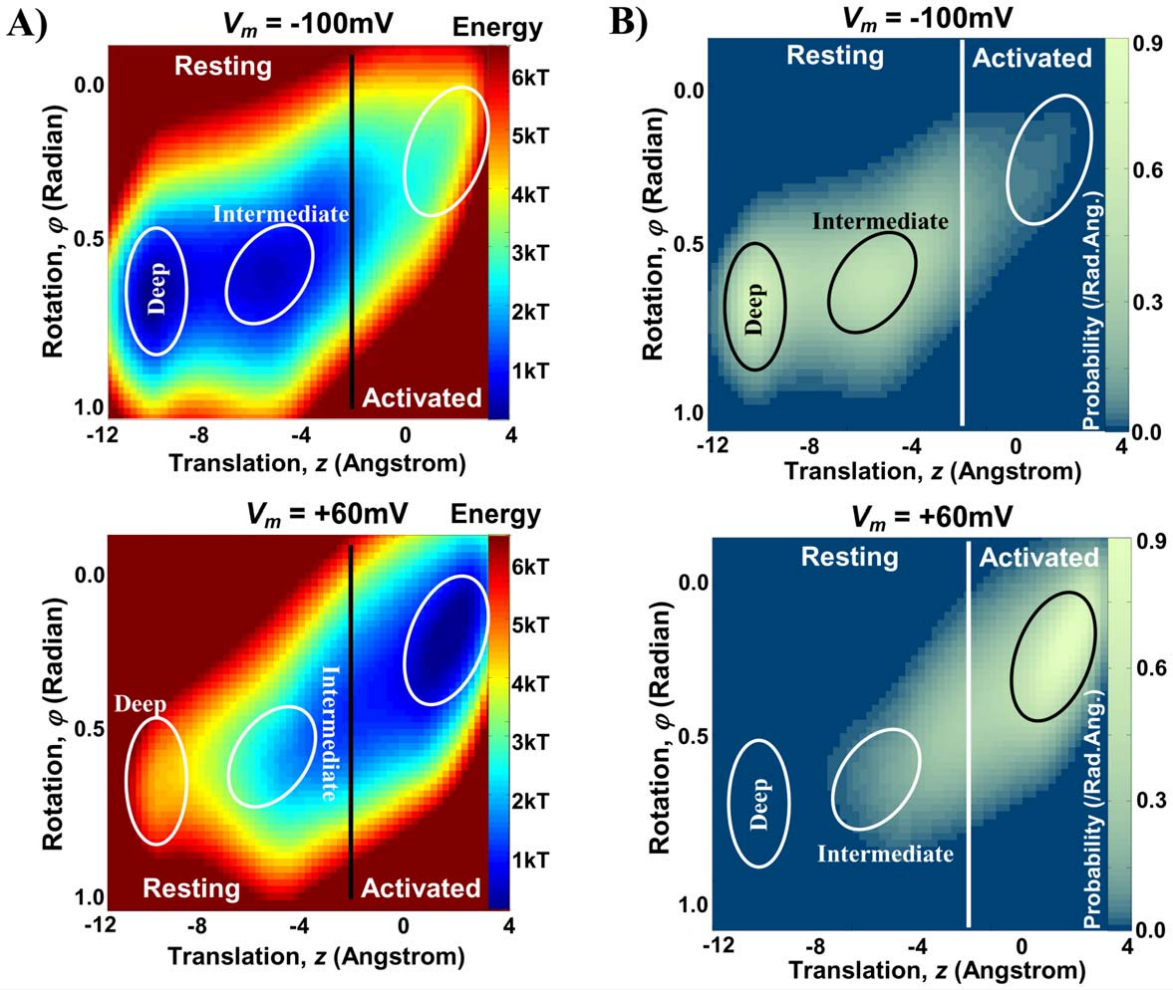
Energy landscapes and their associated steady state distributions (in configuration space) for two different membrane potentials. **A**) Energy landscapes at two different membrane potentials *V_m_* = −100 mV (top) and +60 mV (bottom). Increasing membrane potential shifts minimum energy conformations of the S4–S3 complex toward more outward positions (right side of the energy landscape). Ellipses highlight the energy minima. Vertical black line is the border between resting and activated states. **B**) Steady state distribution of voltage sensors in the configuration space at a particular membrane potential; *V_m_* = −100 mV (top) and +60 mV (bottom). Channels mainly reside in minimum energy conformations (highlighted by the ellipses). Note that the brightness scale is logarithmic. White vertical line marks the border between resting and activated states. In response to a sudden change in membrane potential, voltage sensors distribution varies gradually toward the steady state distribution associated with the new membrane potential (Movies S1 and S2).

The electrostatic interactions between the four voltage sensors of the tetrameric channel are negligible as they are located far from each other. Therefore, we only consider the internal interactions within one voltage sensor plus its interaction with the S5–S6 complex of the neighboring subunit. **Figure 4A** shows the energy landscape for a voltage sensor at two different membrane potentials. Three minima associated with one activated state and two resting states [10], [11], [22] are distinguishable and encircled. As the net gating charge moving with S4–S3 is positive, increasing the membrane potential reduces the potential energy almost linearly in the positive *z* direction (outward S4–S3 conformations). As a consequence, at higher membrane potentials the local minima in the energy landscape shift in the positive z direction.

#### Steady State Distribution of Voltage Sensors

If the membrane has been kept at a constant potential long enough, the distribution of voltage sensors in the configuration space is stationary (steady state condition). **Figure 4B** shows the distribution of voltage sensors among different conformations of configuration space for two membrane potentials, −100 mV and +60 mV. These distributions are calculated using equations (48) and (49) with *C*1 = *kT* and neglecting the second order term. Depolarizing the membrane potential causes the S4–S3 complex to transition to more outward locations. It moves outward up to 12 Angstrom and rotates up to 0.5 radians during this transition.

#### Transient Distribution of Voltage Sensors During Voltage Clamp Test

Transition of the probability distribution from one equilibrated distribution to another in response to a sudden change in membrane potential was computed using equation (43) taking into account the cooperativity of the four subunits during channel opening [15]. Scaling both friction coefficients together only scales the dynamic response in the time domain; it does not affect the shape of the response function in any other way. Therefore, the friction coefficients can be calibrated based on the time scale of the experimentally measured macroscopic current. Using the experimentally recorded currents, the friction coefficient was calibrated to *ζ_z_* = 0.5*10^−3^ kg/s in *z* direction and *ζ_ϕ_* = 12.5*10^−3^ kg.Ang^2^/s in *ϕ* directions. The higher order frictions were neglected. Comparison of these friction coefficients requires transformation to a common dimension. An equivalent translational friction coefficient for rotation about an axis can be derived as:
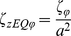
(50)Where *a* is the root mean square distance of the moving residues with respect to the axis of rotation. *a* is about 5 Angstrom in our simulation and we choose *ζ_ϕ_* = *a* ^2^ *ζ_z_* = 25*ζ_z_* so that resistance to motion is similar for the two degrees of freedom.

Equation (43) was solved using the Finite Difference (FD) method to simulate the transition of probability distribution during step depolarization from resting potential of −100 mV to a test potential of +60 mV (activation test), and also during step repolarization from the depolarized potential of +60 mV to the test potential of −100 mV (deactivation test). These transitions and the resultant open probability were visualized for the activation and the deactivation tests in two supplement movies (**Movies S1** and **Movies S2**). By increasing the membrane potential from the resting potential of −100 mV to the test potential of +60 mV, the transition starts with a rapid outward translation and counter clockwise rotation of the voltage sensors to an intermediate conformation, from which they gradually diffuse to a final conformation associated with an additional energy minimum at +60 mV (**Movies S1**).

**Figure 5:**
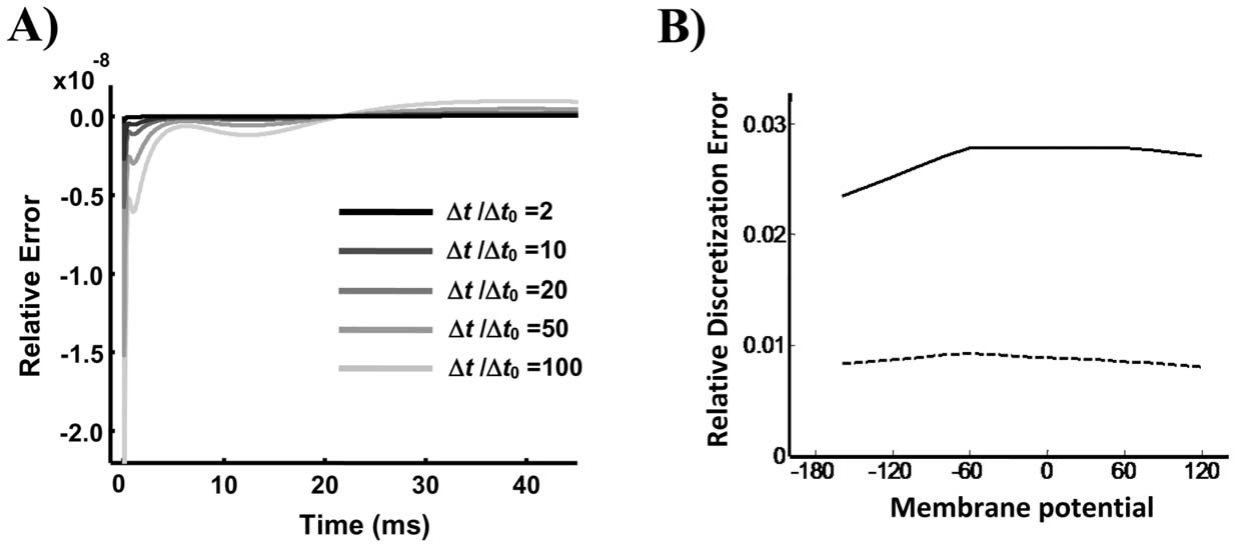
Finite Difference (FD) error in the numerical simulation. **A**) Time variations of the FD error for different integration steps. Each curve shows the integral over the configuration space of the difference between the solution with a given time step (*Δt*) and the solution with the finest time step (*Δt_0_* = 50 ns) as a function of time. FD simulations are reasonably accurate when they converge. *Δt* = 2.5 µs was chosen in the simulations. **B**) Maximum relative discretization error in energy landscapes at different membrane potentials.

To check the accuracy of the FD scheme we computed the difference between 1 and the integral of probability distribution (over the configuration space) during the transition:
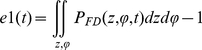
(51)Where 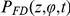 is the FD solution of equation (43). *e*1(*t*) was less than 10^−7^ (<0.00001% error). Additionally, we computed the difference between the probability distribution when the FD simulation approaches steady state (*t* = 1000 ms) and the probability distribution at steady state calculated analytically using equation (48):
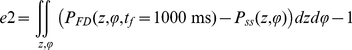
(52)
*e*2 for the voltage clamp test from −100 mV to +60 mV was less than 10^−6^ (less than 0.0001%). To ensure the accuracy of the FD solver, we refined the integration intervals. The FD solver maintained a relative error less that 10^−7^ throughout the gating duration (**Figure 5A**).

Discretization of the configuration space was fine enough to capture all variations in energy smoothly; the maximum relative discretization errors over the energy landscape at all membrane potentials were less than 3% in *z* direction and less than 1% in *θ* direction (**Figure 5B**).

#### Activated and Resting States

At the open state the S4–S3 complex is in an outward position, and during channel closing it moves inward between 7 and 13 Angstroms [19]. The probability distribution at +60 mV shows two high probability regions for voltage sensor conformations. Based on the above experimental observations, the region centered at *z* = 2 and *ϕ* = 0.2 is the activated conformation, that at *z* = −5 and *ϕ* = 0.6 is the intermediate resting conformation and that at *z* = −10 and *ϕ* = 0.7 is the deep resting conformation. An outward conformation of the S4-S3 complex that allows channel opening is termed an activated conformation. The exact border between resting and activated conformations cannot be determined experimentally. We assume that the channel remains in the activated state within 2 Angstrom inward movement of S4-S3 relative to its position in the crystal structure [10], [11], [13] (z = 0 and *ϕ* = 0 in configuration space is associated with the crystal structure conformation). The channel can transition to the open state only if the S4-S3 complexes of all four subunits are in the activated state. The permissive state is the channel state when all four subunits are in their activated states. The cooperative transitions of the channel from the permissive state to the open state and from the open state to the permissive state are modeled by Markovian transitions with transition rates *α* and *β*, respectively.

Transitions to the open state reduce the concentration of voltage sensors in activated states (and consequently in resting states) at depolarized membrane potential due to flux of channels from the permissive state to the open state. Below we explain how to incorporate the cooperativity between the four subunits during transition to the open state in order to calculate the channel open probability. This extends the application of our previous formulation [15] to problems that simulate the conformational changes of the voltage sensor in continuum configuration space (rather than a discrete Markov model).

#### Steady State Open Probability

**Figure 6:**
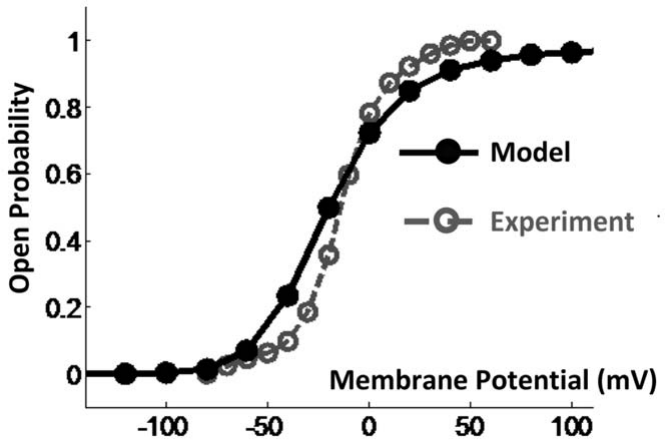
Steady state activation curve computed at several membrane potentials. Solid line shows the simulation results; dashed line is from experimentally measured currents.

At steady state the net transition to and from the open state is zero and the probability distributions of the voltage sensors in configuration space are independent of each other. The open probability can be calculated as [15]:
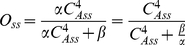
(53)Where *C_Ass_* is the steady state probability of voltage sensors in activated conformations with no transitions to the open state:
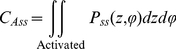
(54)The steady state open probability was calculated at all the test potentials assuming 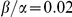. The resultant steady state open probabilities are plotted for different test potentials in **Figure 6**. The simulated curve has the typical S shape dependence on membrane potential. Note that there was no need to incorporate a voltage dependent transition between the permissive and open states to obtain this curve [14].

#### Transient Open Probability

We showed previously how to include the cooperativity of channel opening in computing channel open probability from the probability distribution of its subunits in a discrete Markov model [15]. An analogous approach is also applicable to a continuous distribution in the configuration space. Distribution of voltage sensors in the subunit configuration space changes during channel opening because of 1. redistribution of existing voltage sensors in the subunit configuration space in response to the altered (depolarized) membrane potential, 2. entrance or exit of subunits to or from the subunit configuration space via a net transient flux, *F*(*t*), from or to the open state. Therefore, the transient probability distribution in subunit configuration space, *P_C_*, is the sum of the probability distribution of the subunits that are initially in the subunit configuration space, and the probability distribution of the subunits that enter or exit the configuration space from or to the open state, *P_E_*:
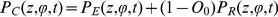
(55)Where *O*_0_ is the initial open probability. *P_R_* shows the distribution of the subunits that are initially in the configuration space (its integral over the subunit configuration space is set to 1). Therefore, probability distribution of the subunits that are initially in the configuration space among all the subunits (taking into account the subunits that are in open state as well) would be (1−*O_0_*)*P_R_*, because only (1−*O_0_*) of the subunits are initially in the subunit configuration space.

The transient flux can be determined using the transition rates *α* and *β*:
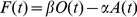
(56)where *A*(*t*) is the probability of the channel occupying the permissive state. As a subunit can transition to the open conformation from any activated conformation, we express the channel open probability, *O*(*t*), in terms of two activated-state probabilities, *U_A_* and *R_A_*, defined as follows [15]:
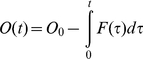
(57)

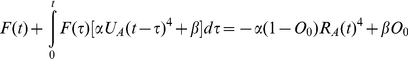
(58)

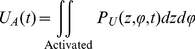
(59)

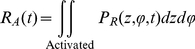
(60)Where *P_U_* is the solution of equation (43) (where there is no net flux to or from the open state) when all subunits are initially in activated conformations distributed proportionally to their final steady state distribution (no subunit is initially in resting conformation), and *U_A_* is probability of activated state under this condition. *R_A_* is the probability of activated state associated with the redistribution of the subunits that are initially in the subunit configuration space. Once the net flux, the open probability and the permissive state probability were computed using the above equations, flux from the open to the permissive state, *βO*(*t*), and from the permissive to the open state, *αA*(*t*) can be determined.

**Figure 7:**
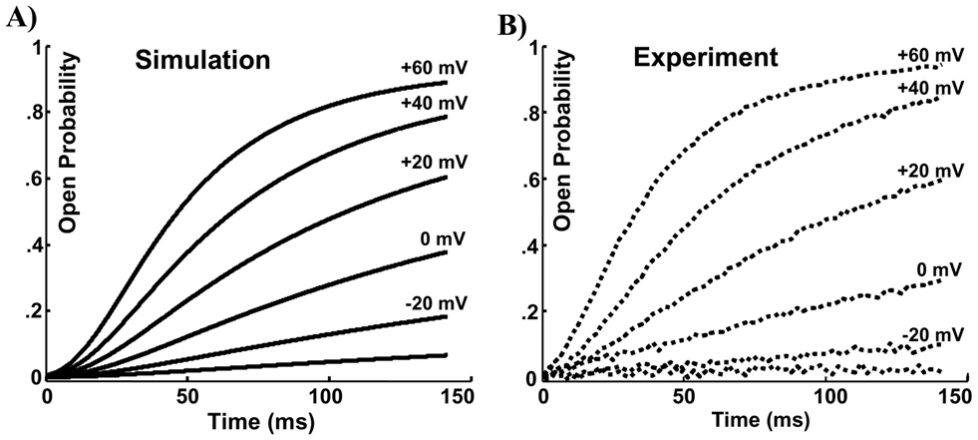
The first 150 ms of the open probability in a series of voltage clamp tests from rest potential of −100 mV to test potentials of −40, −20, 0, 20, 40 and 60 mV. Simulated currents (panel A) closely resemble measured currents (panel B), with slight amplitude difference.

Open probabilities for a series of voltage clamp tests from resting potential of −100 mV to test potentials of −40, −20, 0, 20, 40 and 60 mV were simulated and compared with experimentally recorded currents for the same protocol in **Figure 7**. The simulated transient open probabilities are similar to the experimentally recorded currents; both exhibit a sigmoidal shape (initial delay), biphasic activation (fast then slow) and slower activation at lower test potentials. In these simulations, the central difference method was used for FD simulations and the trapezoidal method was used to compute numerical integrations. The values of *α* and *β* were calibrated to 0.2 /ms and 0.004 /ms. The current traces are similar to those calculated using Monte Carlo simulations and an intermediate Markov model in our previous study [10], [11]. However, the different choice of configuration space in this study resulted in significantly lower energy barriers, so that scaling the membrane potential was not needed in the simulations presented here.

**Figure 8:**
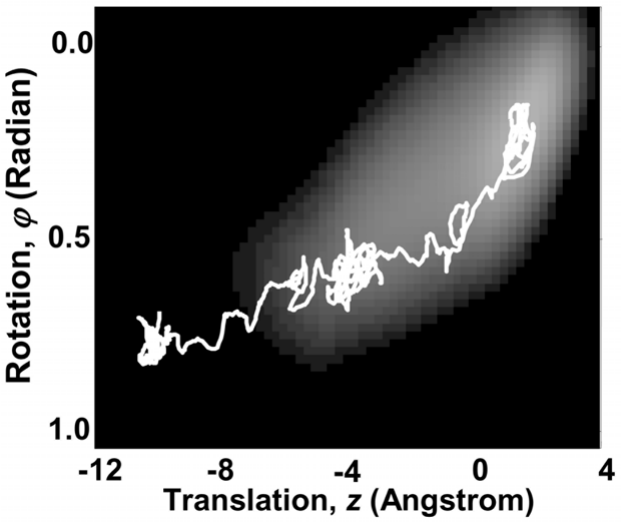
A Motion Trajectory in configuration space in response to a sudden increase in membrane potential from −100 mV to +60 mV. Protein is initially in deep resting state and in response to the change of membrane potential transitions to intermediate resting state, stays there for a while and then transitions to activated state. The associated conformational changes of the voltage sensor are visualized for this motion trajectory in Movie S3.

The trajectory of conformational changes within the configuration space can be estimated using equation (38). **Figure 8** shows one motion trajectory of the average motion in configuration space. Although, the high frequency vibrations of the S4-S3 complex are averaged out, the motion trajectory of gradual conformational changes exhibits a stochastic behavior. Conformational changes of the Kv7.1 voltage sensor along this motion trajectory are animated [23] in **Movie S3**.

## Discussion

Molecular motion of particles under the influence of a conservative force field was analyzed and equation (38) was derived for simulating the average motion trajectory. At the high friction limit, where “*the effect of the Brownian forces on the velocity of the particle* (friction term) *is much larger than that of the external* (conservative) *force*” [24], Langevin equation is reduced to a form similar to equation (38):
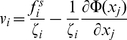
(61)where 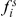 represents the stochastic force associated with thermodynamic collisions. In equation (61) the stochastic velocity term, 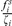, is proportional to the stochastic force, 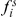. However, from Newton's second law of motion, velocity is proportional to the time integral of the force. This discrepancy is a consequence of eliminating the inertial term from the Langevin equation at the high friction limit. While this approximation is justified between collisions, it is not applicable during collisions. Also, in equation (61) the autocorrelation of stochastic velocity is usually considered a Dirac delta function. However, equation (38) does not make this assumption; in fact, the autocorrelation decreases gradually over *Δt*. As a consequence, the prediction of equation (61) for the stochastic component of the motion is inaccurate. Finally, unlike equation (61), application of equation (38) is not limited to problems where the conservative force is approximated to be constant over the diffusion length (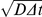). For ion channels that undergo large conformational changes over 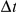 this approximation could be inaccurate. In fact equations (38) and (43) enable the application of Brownian approach for studying the large and gradual conformational changes of proteins.

In our modeling approach, the friction coefficients, *ζ_i_* and *ω_ij_*, are considered as the calibrating parameters. The temperature dependent constant, *C*1, is also a calibrating parameter of the model. However, we chose *C*1 = *kT* based on the Einstein–Smoluchowski relation, derived for ideal gases. We also assumed that the friction coefficient is the same along all degrees of freedom, as we had no reason to include anisotropy. This reduces the calibrating parameters of the modeling framework to a single friction coefficient, *ζ_i_* (i.e. *ζ_z_* for the practical example of this paper).

For simulating the conformational changes of the Kv7.1 ion–channel during gating, the border between the activated and resting conformations and the position of the axis of rotation were calibrated. Note that the position of the axis of rotation is restricted because of steric clashes. The conformation at z = 0 and *ϕ* = 0 in configuration space is associated with the activated state (based on homology with Kv1.2 channel and its known crystal structure in the open state [13]). Channel transitions to the resting state occur by downward translation of S4 (z<0). The location where this state transition occurs (the border line in Figure 4) and the exact position of the axis of rotation were calibrated (along with *α* and *β*, the transition rates between the activated state and the open state) to provide the best fit between the model prediction and the experimental steady state activation curve (Figure 6). Moving the border line shifts the steady state activation curve (about 10 mV for 1 Angstrom dislocation of the border line) but has negligible effect on its slope. Therefore, the slope of this curve can serve as a measure for evaluating the model prediction. This slope represents the sensitivity of the open probability to variations in the membrane potential and is replicated closely by the model. The dielectric constant of the lipid bilayer (as well as the protein) can affect the conservative force field and consequently the simulated open probability. Increasing the dielectric constants of the protein and the lipid bilayer to 8 (from 6) increases the slope of the steady state activation curve by about 6%, while decreasing it to 4 reduces this slope by about 12%.

The transient open probabilities in a series of voltage clamp tests (Figure 7) are also used to evaluate the predictive ability of the model. The friction coefficient *ζ_z_* scales these curves in the time domain but does not change their shapes; it is calibrated to match the time course of activation of the simulated currents to that of the experimental currents.

A methodology for computing the energy landscape and relating it to the macroscopic current was developed in our laboratory and presented by Silva et al. [10]. In that approach, the macroscopic current was estimated using four identical Markov models representing the four channel subunits. Transition rates of the Markov model were assumed to be the reciprocal of the first passage time along an arbitrary path between the minima on the energy landscape. To overcome the complexity associated with the cooperativity between the four channel subunits, open probability was computed through Monte–Carlo simulations. In this paper we use a direct approach for relating the energy landscape to the channel open probability. We derive and solve the equation of motion to find the dynamics of subunit conformational changes in continuum configuration space. We then compute the open probability directly from the dynamics, without using an intermediate Markov model. The cooperativity among subunits is included analytically, avoiding the time consuming Monte–Carlo simulations.

Experimental measurements of the dynamics of gradual conformational changes are extremely difficult to perform, if not impossible. However, for the case of ion–channels these dynamics underly the dynamics of open probability, which can be recorded experimentally as channel conductance (current) under a variety of different conditions. Therefore, a close match between simulated and recorded open probability may be used as verification of appropriate simulations of conformational changes. Consistent with our previous studies [10], [11], [22], the energy landscape has two minima associated with two resting conformations: deep and intermediate. We envision that a more accurate match can be obtained by examining all non overlapping conformations using more than two degrees of freedom. Including more degrees of freedom can also provide more accurate estimates of the protein conformation at resting states [25].

## Methods

The electrostatic energy of the protein was computed at different points of its configuration space using MATLAB codes. Protein and membrane were modeled as continuum dielectrics with dielectric constant of 6, consistent with the range of 2 to 25 determined for lipid bilayers [26], [27], [28] and a range of 2 to 20 determined for membrane proteins [29], [30], [31]. Intra- and extra- cellular electrolyte was modeled as a continuum with dielectric constant of infinity. Interfaces of membrane (and protein) with the abacus environment were modeled by parallel surfaces. The image charge method was used to include the potential energy associated with the surface charge distribution caused by charged protein residues within the membrane [32]. Membrane thickness was assumed to be 30 Angstrom [33]. The protein was placed within the membrane so that the upper membrane surface is right above the E160 residue on the S2 segment. The surface charges were assumed to be distributed within 7 Angstrom from the interface (Debye-Hückel length) with a mean distance of 3 Angstrom. When a charged residue entered the electrolyte environment, the screening effect of this environment was considered by reducing its effective charge to zero within 3 Angstrom.

The governing partial differential equation of the motion (equation (43)) was solved using a central difference FD method and imposing natural boundary conditions. The FD was implemented using MATLAB programming environment and integrated over 50 ns time steps.

## Supporting Information

Figure S1 Velocity trace of a particle computed using the Langevin equation during 10,000 impacts. Panel A) shows the entire trace and panel B) enlarges the small region marked by red ribbon in panel A). Dashed lines in panel B) mark the impact incidents.
(TIF)

Figure S2 Probability density function of the velocity computed using Langevin model (blue curve) compared with its equivalent Maxwell-Boltzmann distribution (red curve). The expectation value of the velocity square is the same for both distributions.
(TIF)

Figure S3 Velocity trace of a particle computed using the model developed in this paper during 10,000 impacts. Panel A) shows the entire trace and panel B) enlarges the small region marked by red ribbon in panel A). Dashed lines in panel B) mark the impact incidents.
(TIF)

Figure S4 Probability density function of the velocity computed using the model developed in this paper (blue curve) compared with its equivalent Maxwell-Boltzmann distribution (red curve). The expectation value of the velocity square is the same for both distributions.
(TIF)

Movie S1 Probability distribution of subunits in configuration space during gating, when the membrane potential increased to +60 mV from a resting potential of −100 mV. Vertical dashed line is the assumed border between resting and activated conformations. The white vertical curve shows the flux within configuration space across this border. The net flux from the permissive state to the open state is shown by the arrow exiting the subunit configuration space at the top right corner. Open state probability is shown (color scale) in the square on the upper right and plotted as a function of time in the right bottom panel.
(WMV)

Movie S2 Probability distribution of subunits in configuration space during gating, when the membrane potential decreased to −100 mV from an activated potential of +60 mV. Vertical dashed line is the assumed border between resting and activated conformations. The white vertical curve shows the flux within configuration space across this border. The net flux from the open state to the permissive state is shown by the arrow exiting the open state toward the subunit configuration space at the top right corner. Open state probability is shown (color scale) in the square on the upper right and plotted as a function of time in the right bottom panel.
(WMV)

Movie S3 A trajectory of conformational changes during gating, when the membrane potential is increased from a resting potential of −100 mV to a depolarized potential of +60 mV. The two orange helical segments are the S4–S3 complex. At −100 mV they are at their most downward location (deep resting). In response to increase of membrane potential to +60 mV they move upward (with slight rotation) to the intermediate resting state. They stay at that state for a while and then move upward along with a noticeable rotation to the activated state. Legend in upper right corner shows the voltage sensor state and in upper left corner the membrane potential.
(WMV)

Text S1 Langevin equation prediction for the velocity distribution. The prediction of Langevin equation for the velocity, in response to a stochastic force with a Gaussian distribution, contradicts the Boltzmann–Maxwell distribution (a Gaussian distribution) for the velocity. Compared to a Gaussian distribution with the same variance, the velocity distribution in Langevin equation has higher densities for velocity magnitudes in close vicinity of zero and for large velocity magnitudes.
(DOC)
