# Supplementary material for: Continuum Molecular Simulation of Large Conformational Changes during Ion–Channel Gating
Source: PLoS One. 2011 May 20;6(5):e20186. doi: 10.1371/journal.pone.0020186 (PMC3098872; doi:10.1371/journal.pone.0020186)
Supplement: Text S1 — Langevin equation prediction for the velocity distribution. The prediction of Langevin equation for the velocity, in response to a stochastic force with a Gaussian distribution, contradicts the Boltzmann–Maxwell distribution (a Gaussian distribution) for the velocity. Compared to a Gaussian distribution with the same variance, the velocity distribution in Langevin equation has higher densities for velocity magnitudes in close vicinity of zero and for large velocity magnitudes. (DOC) [file pone.0020186.s008.doc]

# Continuum Molecular Simulation of Large Conformational Changes during Ion−Channel Gating

# Supplementary Document:

# Langevin Equation Prediction for the Velocity Distribution

by

Ali Nekouzadeh and Yoram Rudy

Cardiac Bioelectricity and Arrhythmia Center and Department of Biomedical Engineering, Washington University in St. Louis, St. Louis, Missouri.

Langevin equation (Equation S1) is a well accepted model for simulating motion at the molecular scale.

$m\frac{\partial v_{i}(x_{j})}{\partial t}=-\frac{\partial\Phi(x_{j})}{\partial x_{i}}-\zeta_{i}v_{i}+f_{i}^{s}=-\frac{\partial\Phi(x_{j})}{\partial x_{i}}-\zeta_{i}v_{i}+\sum_{k} c'_{i}^{k}\delta(t-t_{k})$ (S1)

*m* is the mass of a target particle (ion−channel voltage sensor in the application here), *v_i_* is its velocity along coordinate *x_i_*, Φ is the potential of any existing conservative force field, and the last two terms on the right side of equation represent the stochastic force of thermodynamic collisions on the particle. *t_k_* is a time when another particle collides the target particle and $c'_{i}^{k}$ is the strength of the collision. Because the thermodynamic forces on the target particle have a non−zero mean that is proportional to the target particle velocity (in the opposite direction), they are modeled by a zero mean stochastic force $f_{i}^{s}$, and a friction force $-\zeta_{i}v_{i}$ that introduces the non−zero mean. $\zeta_{i}$ is the friction coefficient.

The stochastic force, $f_{i}^{s}$, is usually assumed to be a Gaussian process with a Dirac delta autocorrelation function. In particular, these assumptions are required in the fluctuation−dissipation theorem[1]. In the following, we consider a simple case where there is no external force field, Φ, and use a computer simulation to determine the probability density function of the velocity that results from Langevin equation. In the absence of external force, Equation S1 reduces to the following first order differential equation:

$m\frac{\partial v_{i}(x_{j})}{\partial t}+\zeta_{i}v_{i}=\sum_{k} c'_{i}^{k}\delta(t-t_{k})$ (S2)

According to this equation, at time *t_k_* the velocity of the particle changes abruptly by $c'_{i}^{k}/m$ and then reduces exponentially with a time constant of $m/\zeta_{i}$ until the next impact. The amplitude of the impact momentum $c'_{i}^{k}/m$ has a zero mean Normal (Gaussian) distribution.

The time between impacts is known to have an exponential distribution and is a random variable in the simulation. For simplicity, we assume that the mean time between impacts is 1. We set the value of $\zeta_{i}/m$ to 1 as well. This represents a case where the mass of the target particle is of the same order as the mass of the colliding particles. Finally, the Normal distribution of $c'_{i}^{k}/m$ is assumed to have a variance of 1. Using these normalized parameters, we computed the velocity of a particle during 10,000 impacts and used that to determine the probability density function of the velocity. **Figure S1** shows the simulated velocity and **Figure S2** shows its probability density function.

-5

-4

-3

-2

-1

0

1

2

3

4

5

-5

-4

-3

-2

-1

0

1

2

3

4

5

**A)**

**B)**

**Figure S1**

Velocity trace of a particle computed using the Langevin equation during 10,000 impacts. Panel A) shows the entire trace and panel B) enlarges the small region marked by red ribbon in panel A). Dashed lines in panel B) mark the impact incidents.

-5

-4

-3

-2

-1

0

1

2

3

4

5

0

0.5

1

1.5

**Figure S2**

Probability density function of the velocity computed using Langevin model (blue curve) compared with its equivalent Maxwell-Boltzmann distribution (red curve). The expectation value of the velocity square is the same for both distributions.

The exponential decay of the velocity magnitude (toward zero) between the collisions (Figure S1 panel B) is an artifact caused by the friction term in the Langevin equation. In the absence of an external force, velocity should remain constant between collisions.

Consistent with the fluctuation−dissipation theorem, the simulation results verified that:

(S3)

where, represents the expectation value. However, the distribution of velocity deviates from its known Maxwell−Boltzmann distribution (red curve in Figure S2).

Simulating the motion using the model developed in this paper generated more realistic results for the velocity trace and its statistical properties as shown in **Figures S3** and **S4**. The velocity remains constants between collisions (Figure 3 panel B) and its distribution coincides with the Maxwell−Boltzmann distribution (Figure 4).

-5

-4

-3

-2

-1

0

1

2

3

4

5

-5

-4

-3

-2

-1

0

1

2

3

4

5

**Figure S3**

Velocity trace of a particle computed using the model developed in this paper during 10,000 impacts. Panel A) shows the entire trace and panel B) enlarges the small region marked by red ribbon in panel A). Dashed lines in panel B) mark the impact incidents.

-5

-4

-3

-2

-1

0

1

2

3

4

5

0

0.5

1

1.5

**Figure S4**

Probability density function of the velocity computed using the model developed in this paper (blue curve) compared with its equivalent Maxwell-Boltzmann distribution (red curve). The expectation value of the velocity square is the same for both distributions.

# References

1. Kubo R (1966) The fluctuation-dissipation theorem Reports on Progress in Physics 29: 255-284.
